# Forecasting Range Shifts in Terrestrial Alpine Insects Under Global Warming

**DOI:** 10.1002/ece3.70810

**Published:** 2025-01-09

**Authors:** Fabio Leonardo Meza‐Joya, Mary Morgan‐Richards, Steven A. Trewick

**Affiliations:** ^1^ Wildlife & Ecology Massey University Palmerston North New Zealand

**Keywords:** adaptive potential, alpine species and ecosystems, correlative and hybrid niche modelling, extinction debt, global warming, range shifts

## Abstract

Anthropogenic planetary heating is disrupting global alpine systems, but our ability to empirically measure and predict responses in alpine species distributions is impaired by a lack of comprehensive data and technical limitations. We conducted a comprehensive, semi‐quantitative review of empirical studies on contemporary range shifts in alpine insects driven by climate heating, drawing attention to methodological issues and potential biotic and abiotic factors influencing variation in responses. We highlight case studies showing how range dynamics may affect standing genetic variation and adaptive potential, and discuss how data integration frameworks can improve forecasts. Although biotic and abiotic factors influence individual species responses, most alpine insects studied so far are shifting to higher elevations. Upslope shifts are often accompanied by range contractions that are expected to diminish species genetic variation and adaptive potential, increasing extinction risk. Endemic species on islands are predicted to be especially vulnerable. Inferences drawn from the responses of alpine insects, also have relevance to species in other montane habitats. Correlative niche modelling is a keystone tool to predict range responses to planetary heating, but its limited ability to consider biological processes underpinning species' responses complicates interpretation. Alpine insects exhibit some potential to respond to rising temperatures via genetic change or phenotypic plasticity. Thus, future efforts should incorporate biological processes by using flexible hybrid niche modelling approaches to enhance the biological realism of predictions. Boosting scientific capability to envisage the future of alpine environments and their associated biota is imperative given that the speed and intensity of heating on high‐mountain ecosystems can surpass our ability to collect the empirical data required to guide effective conservation planning and management decisions.

## Graphical abstract

We conducted a comprehensive, semi‐quantitative review of empirical studies on contemporary range shifts in alpine insects driven by climate heating, drawing attention to methodological issues and potential biotic and abiotic factors influencing variation in response. We highlight case studies showing how range dynamics may affect standing genetic variation and adaptive potential, and discuss how data integration frameworks can improve forecasts.

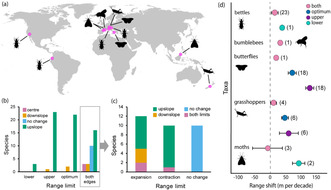

And as the snow melted from the bases of the mountains, the arctic forms would seize on the cleared and thawed ground, always ascending higher and higher, as the warmth increased, whilst their brethren were pursuing their northern journey. (Charles Darwin, The Origin of Species)

## 1 Introduction

**FIGURE 1:**
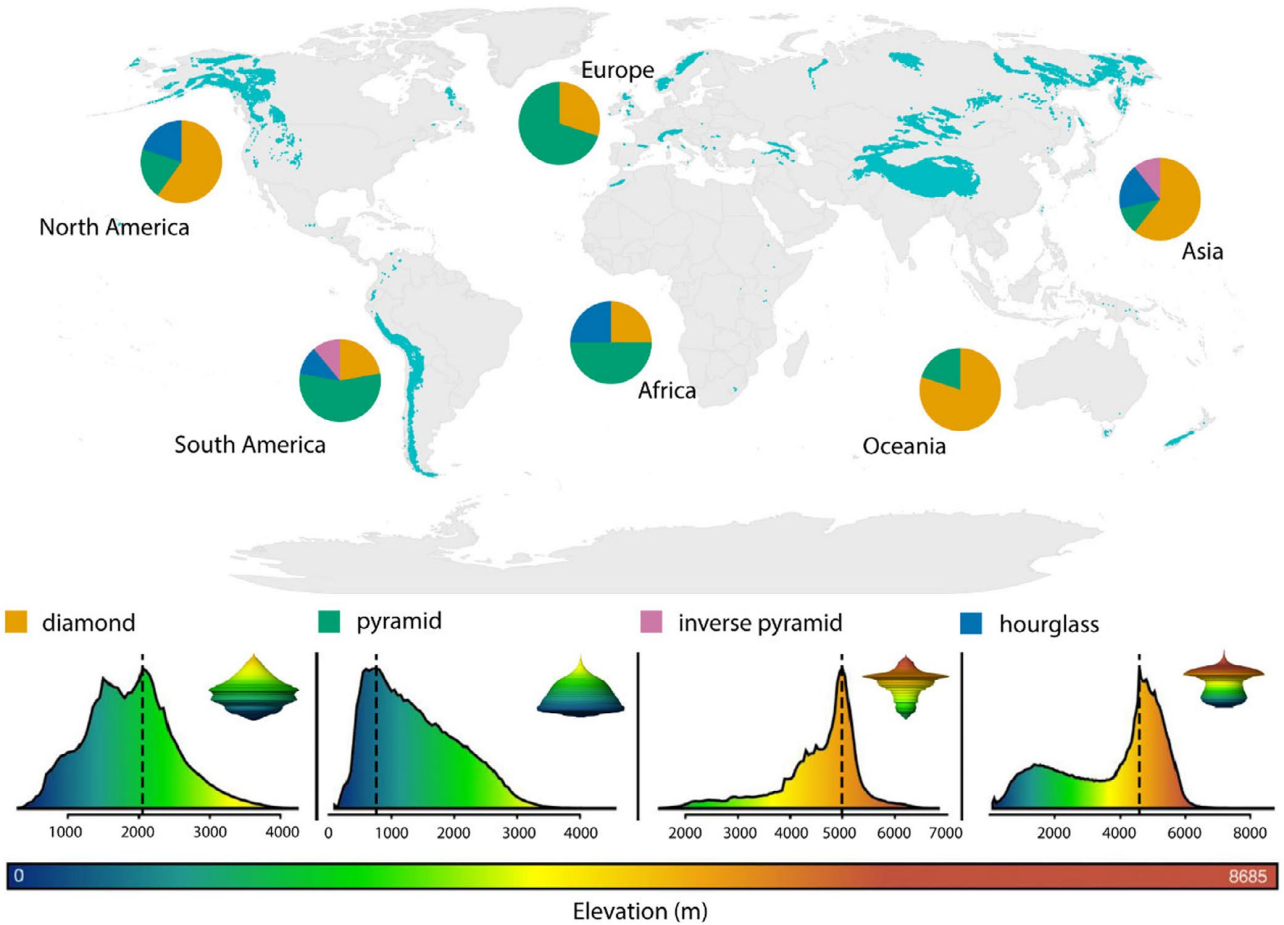
Global distribution of alpine areas (Testolin, Attorre, and Jiménez‐Alfaro 2020) showing their hypsographic classification at the landscape scale based on elevation–area relationships (bottom panels) coloured relative to a maximum elevation of 8685 m (horizontal bar) observed in the Himalayas (modified from Elsen and Tingley 2015). Note that although the alpine area in diamond, hourglass and inverse pyramid mountains can initially increase with elevation depending on their location, it eventually decreases above a certain elevational point (dotted lines). Three‐dimensional model spindles (insets) show the relative surface area available with elevation. Pie charts depict the distribution of each hypsographic class in alpine mountains from six geographic regions: Africa (*n* = 4), Asia (*n* = 28), Europe (*n* = 10), North America (*n* = 10), South America (*n* = 9), and Oceania (*n* = 5). Estimates are based on mountains for which elevation–area relationships are available (*n* = 66; data from Elsen and Tingley 2015).

Alpine zones form on mountains between the natural climatic limit of trees and the permanent snowline (Grabherr, Gottfried, and Pauli 2010; Körner, Paulsen, and Spehn 2011; Testolin, Attorre, and Jiménez‐Alfaro 2020). The elevation of the treeline varies worldwide due primarily to the interaction with latitudinal gradients in solar radiation striking the Earth's surface and local human footprint (He et al. 2023). The elevation where the treeline and alpine zone meet is highest in tropical and subtropical latitudes (reaching more than 4000 m in the Himalayas) and decreases towards the poles (He et al. 2023; Körner 1998; Testolin, Attorre, and Jiménez‐Alfaro 2020). At regional and local scales the position of the tree line reflects topoclimate, species physiology, and human activity (Greenwood and Jump 2014; Harsch et al. 2009; Körner and Paulsen 2004). Oceanic islands generally have lower tree lines than their continental counterparts at the same latitude, reflecting oceanic influence on climates and impoverished woody species diversity (Irl et al. 2016). Wherever the mountain is, and regardless of its shape, the alpine area is attenuated with elevation due to topography (Figure 1). Alpine zones are often interrupted by deep forested valleys or rangelands resulting in a highly patchy habitat, referred to as ‘sky islands’, where populations on separate summits evolve independently from one another (Grabherr, Gottfried, and Pauli 2010; Mayr and Diamond 1976; Pauli and Halloy 2019). Given the analogy between mountaintops and islands, recent research has examined the influence of historical climate‐driven habitat shifts on current patterns of alpine insect biodiversity, using the model of island biogeography (e.g., Marta et al. 2019).

Although globally ubiquitous (Figure 1), alpine ecosystems represent only ~2.6% of the Earth's land surface (Körner, Paulsen, and Spehn 2011; Testolin, Attorre, and Jiménez‐Alfaro 2020) but host a variety of specialised and often endemic biota, including many insects and other arthropods (Chapin and Körner 1995; Mani 1968). Most of the global alpine habitat is in Asia (73%), followed by South America (15%), North America (9%), Europe (2%), while Oceania and Africa together contribute only 1% (Testolin, Attorre, and Jiménez‐Alfaro 2020). Alpine conditions impose significant physiological challenges on life, including low temperatures, high solar radiation, reduced oxygen partial pressure, strong winds, diurnal and seasonal extremes in temperature and water availability, and contracted growth seasons (Dillon, Frazier, and Dudley 2006; Sømme 1989). Furthermore, within alpine zones, complex interactions between climate and topography result in environmental gradients over short distances (De Frenne et al. 2013; Hodkinson 2005; Jump, Mátyás, and Peñuelas 2009), with sharp transitions in vegetation and hydrology (Frazier and Brewington 2020; Grabherr, Gottfried, and Pauli 2010). In response, alpine insects have evolved morphological, phenological, physiological and behavioural strategies such as melanism, brachyptery and freeze tolerance (Sømme 1989).

Alpine habitat is sensitive to climate change, and among the first to respond visibly to rising temperatures brought about by human activity (Steffen et al. 2018). Anthropogenic heating is faster at higher elevations (Pepin et al. 2015), with many wide‐ranging impacts documented (Grabherr, Gottfried, and Pauli 2010; Huss et al. 2017; Pauli and Halloy 2019). Readily apparent are rising treelines and snowlines, and reductions in snowpack depth and endurance (e.g., Cazzolla Gatti et al. 2019; He et al. 2023; Huss et al. 2017; Klein et al. 2016). The advance of treelines to higher elevation reduces opportunities for alpine biota to persist (Beniston 2003; Dirnböck, Essl, and Rabitsch 2011; He et al. 2023; Pauli and Halloy 2019), and this change is emphasised in the tropics where treelines are shifting the fastest (mean of 3.1 m/year; He et al. 2023). Insects and other ectotherms are especially sensitive to the displacement of climatic isoclines because their thermal optima coincide with temperatures that maximise fitness, and individual performance rapidly drops outside the optimal range (Deutsch et al. 2008; Loarie et al. 2009).

Mountains serve as ‘natural laboratories’ with gradients replicated spatially in which biotic responses to geophysical conditions can be investigated (Figure 1), and have stimulated biodiversity research for centuries (Körner 2007; McCain and Garfinkel 2021). Range shifting is considered a major response of insect species to global warming (McCain and Garfinkel 2021; Rumpf et al. 2019). In mountain ecosystems, climate heating is promoting upslope shifts of insect species, but patterns of movement are complex (McCain and Garfinkel 2021) and shift trends for alpine species have yet to be fully explored. To date, range shifting in alpine species is best documented by predictive modelling, an approach that has gained momentum with the advancement of statistical algorithms (McCain and Garfinkel 2021; Neupane, Larsen, and Ries 2024). However, direct evidence of the impacts of rising temperatures on species elevational ranges come from empirical studies comparing historical data (pre‐1980s) with more recent data gathered during the ongoing period of rapid climate warming (McCain and Garfinkel 2021). Although one previous review broadly addressed possible effects of climate change on high elevation insect communities (Shah et al. 2020), many knowledge gaps remain, including the geographic and taxonomic spread of empirical range shifts in alpine inhabitants.

Here, we conducted a comprehensive, semi‐quantitative review of empirical studies on contemporary climate‐driven range shifts in alpine insects, one of the most ubiquitous and diverse animal groups in terrestrial alpine ecosystems worldwide (Hein et al. 2019). We draw attention to factors with potential to influence range shift rates and outline how range dynamics may affect standing genetic variation and adaptive potential. We discuss the mechanisms underlaying range shifts and illustrate how integrating multiple data sources and analytical tools can help to overcome several methodological limitations to improve forecasts. We propose that predictive modelling should make use of available data, even if limited, while incorporating biological processes into flexible, hybrid niche modelling approaches to enhance the biological realism of predictions.

## 2 The Legacy of Past Climates on Alpine Biogeography

Climate is a fundamental force shaping biological composition apparent in the fossil record by large‐scale migration, extinction and speciation (Eyles 2008; Huntley and Webb 1989; Stanley 1988), and evident from contemporary observations of species ranges and life cycles that respond to changing climates (Walther et al. 2002; Parmesan 2006; Freeman et al. 2018). Plio‐Pleistocene climate cycles shaped much of modern biogeography as periods of cooling and glacial advance are imprinted in the population genetic structure of many warm‐adapted species and indicate changes in habitat availability promoting range contractions and extinctions (Davis and Shaw 2001; Hewitt 1999, 2004; Taberlet et al. 1998; Riddle and Honeycutt 1990). In contrast, cold‐adapted taxa experienced population and range expansions in response to these same conditions (Birks 2008; Endo et al. 2015; Galbreath, Hafner, and Zamudio 2009; Hewitt 2004; Trewick, Wallis, and Morgan‐Richards 2000). At the start of the current interglacial period, many warm‐adapted species underwent population expansion recolonising higher latitudes as ice sheets receded, and forests advanced in montane areas at the expense of alpine habitat (reviewed in Hewitt 2004). Thus, cold‐adapted taxa were pushed to higher elevation and latitude, experiencing range contraction and population demise (e.g., Ikeda 2022; Muellner‐Riehl 2019; Trewick, Wallis, and Morgan‐Richards 2011) that is evident from the paleontological record (e.g., Lister and Sher 2001; Stuart et al. 2004). Range contraction and fragmentation of high elevation habitat resulting from anthropogenic climate warming continues at an accelerated rate (e.g., Carmelet‐Rescan et al. 2021; Endo et al. 2015; Hewitt 2004; Meza‐Joya et al. 2023; Trewick, Wallis, and Morgan‐Richards 2000).

Genetic variation provides populations with the adaptive potential to respond to new conditions such as climate change (Frankham 2003; Martin et al. 2023; Pauls et al. 2013), but the current distribution of genetic variation reflects the extent of past population reduction and isolation (Meza‐Joya et al. 2023). As in other alpine organisms, postglacial range contractions in alpine insects left signatures of high, spatially structured, genetic diversity from larger ancestral populations indicative of demographic demise (e.g., Carmelet‐Rescan et al. 2021; Endo et al. 2015; Hewitt 2004; Meza‐Joya et al. 2023; Trewick, Wallis, and Morgan‐Richards 2000). Topography, landscape configuration and species dispersal abilities among other factors, are known to have influenced past range shifts (Trewick, Wallis, and Morgan‐Richards 2000; Meza‐Joya et al. 2023), and thus standing genetic diversity and structuring. Simulations have stressed the role of range contractions in eroding neutral (with respect to natural selection) genetic diversity (Arenas et al. 2012; Rogan et al. 2023), indicating lineages and alleles at the receding edge of a population are more likely to disappear (Cobben et al. 2011; Arenas et al. 2012). This is in line with inferences from genetic data and niche modelling in alpine insects. For example, range contractions, driven by climate warming, are expected to result in loss of genetic diversity and intraspecific lineages in alpine *Sigaus* grasshoppers from New Zealand (Meza‐Joya et al. 2023). While no empirical accounts of this phenomenon exist for terrestrial alpine insects, there are examples in other alpine organisms, including aquatic stoneflies (Jordan et al. 2016), mammals (Rubidge et al. 2012) and plants (Alsos et al. 2009). The ability of species and populations to keep pace with future climate change depends on their adaptive potential, and so may be constrained by erosion of variation in this way (Frankham 2003; Martin et al. 2023; Pauls et al. 2013).

## 3 Contemporary Range Shifts in Alpine Insects

Net global warming is pushing most montane insects upslope (reviewed in McCain and Garfinkel 2021). Populations residing at elevations where alpine conditions prevail and the effects of warming are magnified (Pepin et al. 2015), are expected to track colder conditions upward (McCain and Garfinkel 2021; Shah et al. 2020). On the other hand, the elevational limits of many insect species are determined by their capacity to handle temperature variance (i.e., thermal tolerance), and variance is greatest at higher elevations (Hodkinson 2005; Sømme 1989). Thus, high‐elevation insect species with wide thermal tolerance might not need to move far to track their thermal niche (Mamantov et al. 2021). We use a systematic review of empirical studies that compare historical and contemporary elevational data for alpine insects to elucidate range shift responses to global warming. We sought peer‐reviewed published literature (up to October 2024) in two major electronic databases (Google Scholar and Web of Science) using the keywords and Boolean operators: “alpine insects” AND “range shifts” (OR distributional change) AND “global warming” (OR “climate change”). A second search to refine the initial search used: “alpine insects” AND “range contractions” (OR range expansion) AND “global warming” (OR “climate change”). From the results we focused on studies that (1) presented empirical data on species‐level elevational shifts, (2) included data not reported in the original sources, and (3) reported data from online databases. We restricted our review to studies spanning more than 10 years to avoid timeframes with minimal or no warming trends.

In addition to the literature search we included empirical range shift data for four alpine grasshoppers (Orthoptera: Acrididae) in the genus *Sigaus* (Trewick, Koot, and Morgan‐Richards 2023), endemic to New Zealand's Southern Alps: *S. australis* , *S. nitidus* , *S. nivalis* , and *S. villosus* . For this we resurveyed two elevation transects in Craigieburn Forest Park, South Island, New Zealand, after an interval of 52 years. These transects extend from near the timberline to the ridges of the mountains, and were first sampled during the summer season of 1968–1969 by Watson (1970). Eighteen plots were sampled in the original surveys, 10 in Alan's Basin (−43.122, 171.688; WGS84) and eight in Camp Stream (−43.129, 171.704; WGS84). However, four plots were excluded here: two from Camp Stream due to major land‐use change (experimental pine planting) and two from Alan's Basin due to missing geographic information and differences in sampling areas. Watson (1970) provided aerial imagery of the sampling plot locations, along with data on grasshopper occurrences, surveyed areas (10 × 20 m plots), elevation, aspect, and slope for each transect. We uploaded aerial images to Google Earth Pro (Google Inc.) to relocate the plots, accounting for elevation, aspect, and slope features (with a 30 m location uncertainty), and repeated the elevational‐transect surveys during the summer seasons of 2021–2024. The field methods and search area (200 m^2^) of the original surveys were replicated to allow direct comparisons. Briefly, the occurrence of grasshopper species inside each plot was recorded as we walked through, and was coded as absent (0) or present (1), and then the number of grasshopper species per plot was estimated. Species were identified following Bigelow (1967).

**FIGURE 2:**
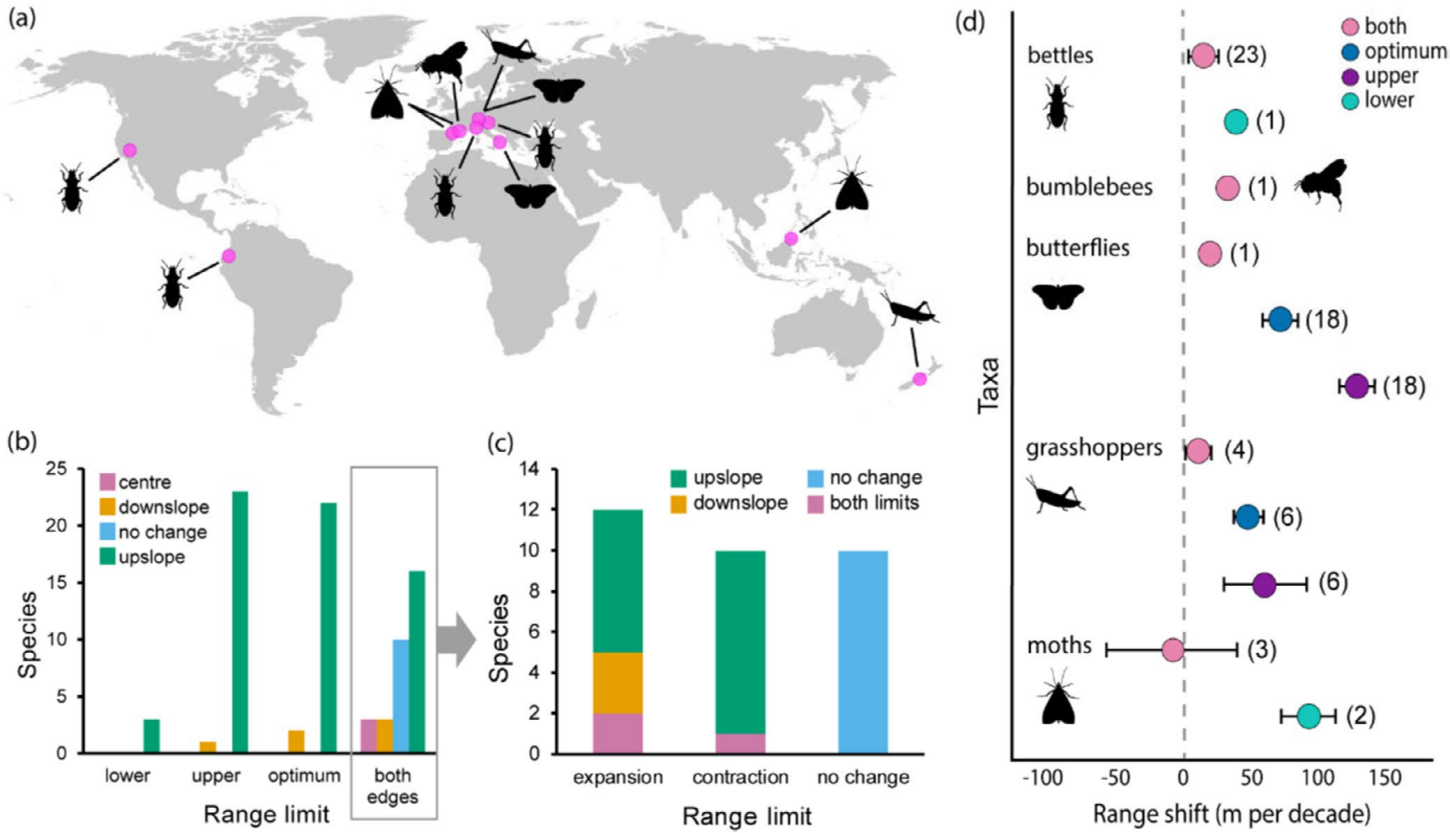
Geographic distribution of alpine beetles, bumblebees, butterflies, moths and grasshoppers used in analyses (a). Most range shifts are upslope regardless of the examined range limit (b). Estimates based on single limit data (*n* = 83) are upslope or downslope responses, while estimates based on complete range data (*n* = 32) revealed six response types (c). Comparisons of mean elevational range shift per taxon indicate mostly positive trends. Values correspond to the mean range shift per taxon (dots coloured by the range limit examined) with standard error bars and number of species considered in parentheses (d). Range shift values are as reported in the original study or estimated based on published raw data (estimations based on distinct range limits are not directly comparable). Data are available online in Tables S1 and S2. All silhouettes but grasshoppers were created with BioRender (https://www.biorender.com).

Our electronic database search strategies revealed only 25 papers that provided empirical data on distributional changes for alpine insect species (Table S1), a shortfall that requires urgent attention. Most of these studies (60%) focused on montane gradients sampling a single alpine site (e.g., Bonelli et al. 2021; Forister et al. 2010; Franzén and Öckinger 2012; Halsch et al. 2021) or compiled insect data from other studies (e.g., Freeman et al. 2021; Lenoir et al. 2020; Mamantov et al. 2021; McCain and Garfinkel 2021; Rubenstein et al. 2023; Rumpf et al. 2019). We identified 10 studies reporting comprehensive empirical data for 83 alpine species and used these in our analyses (Chen et al. 2011; Dahlhoff et al. 2019; Dieker et al. 2011; Marshall et al. 2020; Menéndez et al. 2014; Moret et al. 2016; Panza and Gobbi 2022; Pizzolotto et al. 2014; Scalercio et al. 2014; Vitasse et al. 2021). Each of these studies used historical baseline data; thus, range shift responses used distinct range limits (Table S2): lower, upper, both (lower and upper) or optimum (i.e., elevation of maximum abundance). Each study has limited geographic and taxonomic coverage with most species being European Lepidoptera (Figure 2a), which is in striking contrast to the ubiquity and diversity of alpine insects. These studies (or data included therein) along with our own empirical data (Table S2) revealed that most studied species (77%) display upward range shifts (Figure 2b).

Robust inference of range shifts should include tests of both lower and upper range limits as estimates based on single limits (either lower, upper or optimum) only inform partial responses that impede meaningful risk assessment (Chen et al. 2011; Rumpf et al. 2019; McCain and Garfinkel 2021). To account for this, we reanalysed the data for a subset of 32 species for which complete ranges were available (Figure 2c) and found six different responses. Notably, upslope shifts were often accompanied by range contractions (28% of the species), in agreement with predictions from ecological niche modelling (e.g., Bonifacino et al. 2022; Koot, Morgan‐Richards, and Trewick 2022; Múrria et al. 2020; Poloni et al. 2022) and linear regression (e.g., Biella et al. 2017; Bonifacino et al. 2022; Rödder et al. 2021). Despite this, upslope shifts led to range expansion in a number of species (22%) suggesting that their trailing edges had not yet tracked warming (McCain and Garfinkel 2021) or that factors other than temperature (e.g., biotic interactions) are important at the lower limit (Alexander, Diez, and Levine 2015; Rumpf et al. 2019). Apart from upslope trends, range stasis (i.e., no change at either limit) was common (31% of the species), particularly among carabid beetles and acridid grasshoppers with low dispersal ability (Moret et al. 2016; Meza‐Joya et al. 2023).

Unexpected responses to warming include downslope expansions in the lower limit (9%), range expansions in both limits (6%), and contractions of both limits (3%), but most taxonomic groups showed positive trends in mean elevational range shifts (Figure 2d). European butterflies (Lepidoptera) showed the strongest positive trends in response to global warming, but there was considerable variation within and among the studied taxon groups such as geometrid moths in Borneo that showed both positive and negative trends. There are many possible explanations for this variability. Methodological issues (e.g., detection errors due to under‐sampling or interannual population variation) and lagged responses might have contributed (McCain and Garfinkel 2021; Rumpf et al. 2019), but range responses may also reflect the phenotypic and genetic diversity of individual species' variance in temperature change across their ranges (Bellard et al. 2012; Moritz and Agudo 2013; Rinnan and Lawler 2019). For example, non‐identical range shifts among alpine *Sigaus* grasshoppers in New Zealand signal differences in niche specialisation and ecology, and also uneven climate departures across their ranges under future warming (Meza‐Joya et al. 2023; Meza‐Joya, Morgan‐Richards, and Trewick 2024). Species may also display range change as a direct consequence of land use change (Lenoir et al. 2010; Rumpf et al. 2019). For instance, contemporary changes in grazing intensities are thought to underpin the upslope movement of alpine *Zygaena* moths in the Pyrenees (Dieker et al. 2011), while historical changes in plant communities following human‐mediated fires allowed some New Zealand alpine grasshoppers to expand their ranges downward (Halloy and Mark 2003). Inferences drawn from the responses of alpine insects, also have relevance to species in other montane habitats and systems with less steep environmental gradients (e.g., McCain and Garfinkel 2021).

**FIGURE 3:**
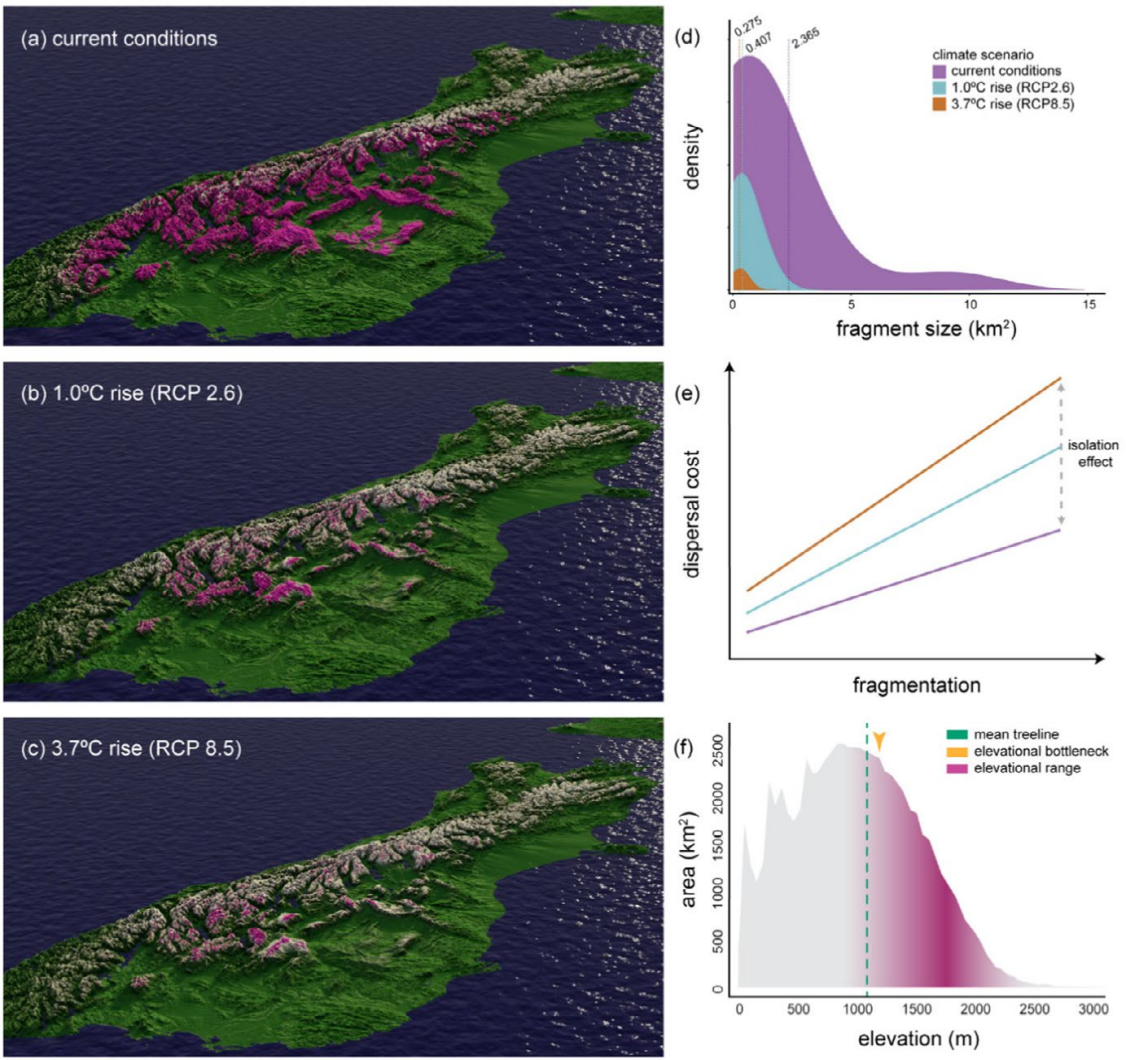
Oblique 3D projections (a–c) of Kā Tiritiri o te Moana (Southern Alps) of Aotearoa‐New Zealand, depicting inferred shifts in the suitable niche space for the endemic, flightless, alpine grasshopper *Sigaus australis* under two future climate scenarios (Koot, Morgan‐Richards, and Trewick 2022; Meza‐Joya et al. 2023). Predicted habitat fragmentation based on density distributions of suitable patches (> 0.1 km^2^) under current conditions and future scenarios (d). Vertical dashed lines indicate means of each distribution with their values (modified from Koot, Morgan‐Richards, and Trewick 2022). Hypothetical relationships between dispersal cost and habitat fragmentation: Dispersal costs is predicted to increase due to climate‐driven habitat fragmentation under future warming scenarios (e). The steeper the slopes, the more limited dispersal opportunities that is, increasing isolation with respect to current conditions. Elevation–area relationship for the Southern Alps (Elsen and Tingley 2015) coloured relative to the inferred current elevational range of *S. australis* (Meza‐Joya et al. 2023; f). The mean treeline position at 1060 ± 173 m (Rita et al. 2023) and the elevational bottleneck at 1160 m (Elsen and Tingley 2015) above which area decrease monotonically with elevation are indicated. Map projection: NZGD2000, with vertical scale emphasised for clarity.

Factors other than warming and land use change (e.g., precipitation, humidity, biotic interactions, food resources, oxygen availability) might also be important drivers of range change (McCain and Garfinkel 2021; Rumpf et al. 2019). For example, the obligate dependency of alpine butterflies (Lepidoptera) on host plants in the German Alps seems to represent an important limiting factor for the establishment of these species towards the cold, upper end of the environmental gradient (Kerner et al. 2023). Dispersal attributes (Marta et al. 2019) in relation to topography (Elsen and Tingley 2015) can be influential, with are expected to result in range contraction among flightless alpine *Sigaus* grasshoppers on remote islands such as New Zealand (Figure 3). In this case, unsuitable habitat bounding both islands and mountains, and a narrow elevational span of suitable habitat (i.e., lower topography relative to continental mountains and area declines with elevation) would magnify the “summit trap” effect that operates on mountains (Dirnböck, Essl, and Rabitsch 2011; Sauer et al. 2011) and lead to local extinctions as species are pushed off mountaintops (Koot, Morgan‐Richards, and Trewick 2022; Meza‐Joya et al. 2023; Meza‐Joya, Morgan‐Richards, and Trewick 2024). This phenomenon is epitomised by alpine insect species that have disappeared from some mountaintops during the last century of warming, such as the European butterfly *Colias phicomone*, the mountain moth *Zygaena exulans*, and the ground beetle *Nebria germarii* (Bonelli et al. 2021; Dieker et al. 2011; Panza and Gobbi 2022; Pizzolotto et al. 2014).

## 4 Mechanisms for Shifts Driven by Climate Warming

**FIGURE 4:**
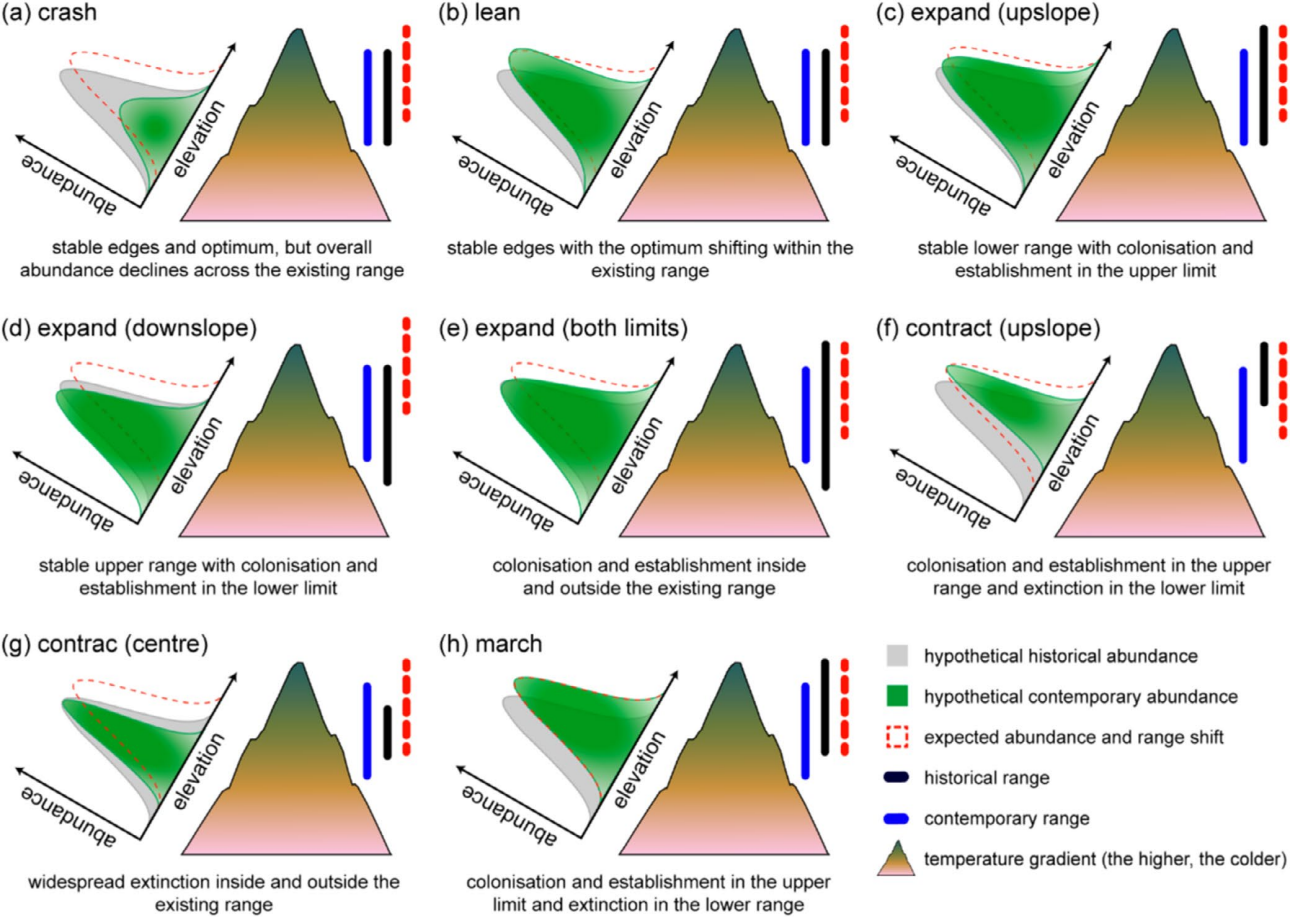
Conceptual representation of distinct range dynamics in alpine insects and their positions in a bi‐dimensional space defined by species' persistence and dispersal rates. These range shifts can inform whether species display low climate tracking but high persistence (a, b), high persistence rate and high movement rate (c–e), low climate tracking and low persistence (f, g), and (h) high climate tracking but low persistence (see Lenoir and Svenning 2015). Note that crash and lean range‐shifts are particularly relevant for those species with stable ranges in our dataset (31% of the species), which lack empirical abundances. Retracting and crashing species will be most at risk of extinction, while expanding and marching species will be less threatened (Lenoir and Svenning 2015). Darker areas in densities curves after climate change indicate higher abundances. These range‐shift types are not mutually exclusive and could occur in various combinations resulting from changes in population growth, establishment, decline, and/or demise.

Range shifting is perhaps the best‐documented insect response to global warming (McCain and Garfinkel 2021), yet the mechanisms underlying these distributional responses are poorly understood (see Diamond 2018). Organisms shift their ranges to stay in quasi‐equilibrium with the climate envelope they are adapted to (Bellard et al. 2012), but doing so can involve both plastic and adaptative evolutionary responses (Atkins and Travis 2010; Diamond 2018; Martin et al. 2023). Initial responses buffering the effects of climate heating and other changes in the environment, are commonly identified as phenotypic plasticity (Merilä and Hendry 2014; Parmesan 2006), but adaption of a population (i.e., trait change by selection on heritable allelic variants) is the only way that the bounds of plasticity can change (Hoffmann and Sgrò 2011; Kinzner et al. 2019; Martin et al. 2023). Disentangling one from the other is challenging as they are not mutually exclusive, can interact, work additively, and plasticity can either impede or promote evolutionary change (Chevin and Hoffmann 2017; Diamond 2018; Martin et al. 2023). Therefore, plasticity and adaptive evolution are key for range dynamics and may dictate whether alpine insects shift their ranges to track suitable climates or persist in place under warming (Figure 4).

Range dynamics may be strongly influenced by range‐limiting traits (Diamond 2018) although empirical data for alpine insects remains scarce so far. Alpine *Grylloblatta* ice‐crawlers in the Cascade mountains (USA) have minimal physiological plasticity in terms of temperature (narrow thermal limits) that are expected to prevent upslope shifts and eventually lead to extensive range contractions (Schoville et al. 2015). To our knowledge, no studies have directly compared thermal acclimation between tropical and temperate alpine terrestrial insects. Yet, acclimation experiments indicate that mayflies (Ephemeroptera) from more thermally variable environments, such as temperate and high elevation areas, display greater acclimation ability than their tropical and low elevation counterparts (Shah, Funk, and Ghalambor 2017), and such physiological differences may have implications for range shifting. Limited adaptive potential for heat resistance in the alpine fly *Drosophila nigrosparsa* from the European Alps is expected to echo this pattern (Kinzner et al. 2019). Likewise, alpine acridid grasshoppers from the Bavarian Alps (Germany) display narrow climatic niches, which is expected to promote their retreat to cold microclimate sites as temperatures rise (König et al. 2024). Tracking of suitable conditions depends on a species capacity for dispersal and can be a key determinant of range shift in alpine insects where flight loss is common (Buckley, Hoare, and Leschen 2022; Sømme 1989). Where wingspan is a plastic traits such as in alpine bumblebees (Apidae) of the Cantabrian mountains (Spain), uphill shift of long‐winged individuals at lower elevations may not be matched by dispersal of the shorter winged individuals at higher elevations (Laiolo, Illera, and Obeso 2023). Likewise, lower partial pressure of oxygen at higher elevations might constrain the leaf beetle *Chrysomela aeneicollis* in the Sierra Nevada (USA) moving upwards by hypoxia that reduces larval performance (Dahlhoff et al. 2019).

Species specific capacity for response may be overshadowed by species interactions as asynchronous range shifts with climate can alter evolutionary trajectories (Alexander, Diez, and Levine 2015; Urban et al. 2016). In the German Alps, butterfly‐host plant interactions mediate range dynamics, as butterflies can move upslope faster than their existing host plants, and this mismatch might limit the ability of these insects to establish at higher elevations if butterflies do not have existing plastic capacity to utilise or adapt to alternative host plants (Kerner et al. 2023). Likewise, recent theorical (e.g., Thompson and Fronhofer 2019) and empirical work (e.g., Alexander, Diez, and Levine 2015) emphasised that shifts in the competitive environment can greatly influence range dynamics, and this might be particularly relevant for diverse alpine communities with numerous species interactions. We are aware of no study exploring the effects of such competitive changes on alpine insect range dynamics, but experimental translocation of alpine plants in the French (Choler, Michalet, and Callaway 2001) and Swiss Alps (Alexander, Diez, and Levine 2015) shows that novel competition could increase with climate warming if species from lower elevations move up the gradient. Differences in species evolutionary potential may also allow faster‐adapting species to persist in their current ranges, and prevent by competition, other taxa from shifting to track environmental change (Thompson and Fronhofer 2019). Notably, all these studies highlight the need to consider ecological and evolutionary processes together when forecasting range shifts.

Where dispersal permits, an alternative to genetic decline is genetic rescue through influx of low elevation alleles into small, inbred, high‐elevation populations (or species), or warming‐induced speciation by the divergence of existing populations into two (or more) distinct species adapted to local climates (Shah et al. 2020). For example, hybrid *Lycaiedes* butterflies evolve behavioural and ecological traits that allowed for persistence in the environmentally extreme alpine habitat and reproductively isolate these populations from their parental species (Gompert et al. 2006). However, environmental change can also cause time‐delayed extinctions, known as extinction debt, as in European butterflies and burnet moths (Krauss et al. 2010). Here, long‐lived species can persist in degraded habitats for longer than short‐lived ones, but are nevertheless unlikely to maintain viable populations. We know of no experimental or observational data in alpine insect populations connecting climate change to extinction debts. Although not directly due to climate change, range shift of New Zealand alpine grasshoppers into exotic anthropogenic habitat (Halloy and Mark 2003) is expected to yield small lowland populations with low genetic diversity that might experience lag‐extinction effects (Meza‐Joya et al. 2023; Meza‐Joya, Morgan‐Richards, and Trewick 2024).

## 5 Species Range Shift Forecast and Uncertainty

Ecological niche modelling (ENM) is the keystone tool for predicting range responses to global warming, particularly models using a purely correlative approach that directly links species location data (i.e., realised niche) and climatic predictors (Briscoe et al. 2019; Diamond 2018; Dormann et al. 2012; Wiens et al. 2009). Correlative ENMs are data‐friendly, relying on either presence‐only or presence‐(pseudo)absence data. However, they are limited in their predictive capacity and transferability to novel conditions, and ignore key biotic mechanisms (Table 1) that set species range boundaries (Briscoe et al. 2019; Dormann et al. 2012; Urban et al. 2016; Wiens et al. 2009). Like any mathematical model, ENMs make assumptions, both biological (e.g., equilibrium and habitat saturation) and statistical (e.g., even sampling and full detection) that are rarely met in real world datasets. They also incorporate uncertainties related to scale choice and input data accuracy, including from physical models used to build climate layers for projections (Briscoe et al. 2019; Dormann et al. 2012; Neupane, Larsen, and Ries 2024; Wiens et al. 2009). Critically, the circular nature of the modelling process precludes hypothesis testing (i.e., the modelled causal relationship), as the data used for hypothesis formulation cannot also be used for testing (Dormann et al. 2012).

**TABLE 1:** Correlative niche modelling often ignore key biological mechanism(s) that set species range boundaries.

Biological mechanism	(a) Detail of biological mechanisms	(b) Incorporation in model predictions
Physiology	Physiological processes reflecting tolerance or stress to climate settings can influence species' realised niches, particularly if physiology sets range boundaries (Helmuth, Kingsolver, and Carrington 2005)	The limited heat tolerance of an alpine fly species is expected to result in a generalised loss of its range under future warming scenarios (Kinzner et al. 2019)
Demography and phenology	Demographic (birth, death, migration) and phenological traits (timing of life history events) play critical roles in shaping species' realised niches (Ponti and Sannolo 2023)	We know no study linking niche modelling and phenology in alpine insects, but this approach has proved phenology dictates future range shifts in widespread grasshoppers (Lemoine 2021)
Evolutionary potential and local adaptation	Evolutionary potential can help species counter stressful conditions or realise ecological opportunities arising from climate change (Hoffmann and Sgrò 2011)	Limited evolutionary potential in an alpine species (fly), is likely to lead to the loss of most of its range under warming scenarios (Kinzner et al. 2019)
Biotic interactions	Interacting with other species can shape species' realised niches depending on the direction (positive or negative) and strength of such interactions (Wiens et al. 2009)	Range shifts in the co‐occurrence of interacting flea beetles and host plants will alter interactions and potentially restructure insect communities (Cerasoli et al. 2020)
Dispersal and colonisation	Dispersal constraints (e.g., limited dispersal ability or the physical environment) may restrict a species' realised niche, particularly when suitable patches are isolated (Alexander, Diez, and Levine 2015)	Range contractions of flightless grasshoppers will be intensified due to limited capacity of local populations to track suitable conditions (Koot, Morgan‐Richards, and Trewick 2022)
Responses to environmental variation	Species specific differences in sensitivity and exposure to climate variation at relevant spatiotemporal scales can influence species' realised niches (Urban et al. 2016)	Range shifts of flightless grasshoppers will depend on species‐specific sensitivities and population exposures to climate variation across their ranges (Meza‐Joya et al. 2023)

*Note:* Investigating such biological processes is needed to improved forecasts of alpine biodiversity, and this can be attained by integrating correlative model forecasts with multiple data sources and analytical tools. Here, we provide examples of literature considering particular biological mechanisms (a), and exemplars of studies that investigate biological mechanisms to improve model forecast for alpine insects (b).

Several methods to boost range shift inferences from correlative models have been applied to alpine insects, including ensemble forecasting where multiple projections are integrated (e.g., Biella et al. 2017; Carmelet‐Rescan et al. 2021; Cerasoli et al. 2020; Koot, Morgan‐Richards, and Trewick 2022) and statistical approaches to reduce overfitting and consider extrapolation risks (e.g., Meza‐Joya et al. 2023; Meza‐Joya, Morgan‐Richards, and Trewick 2024). Other analytical approaches can complement correlative modelling to help detect the influence of biological processes on range shift predictions (Table 1). These include: phylogeography and historical demography to infer range shifts in response to past climates and geophysical events (Brunetti et al. 2019; Carmelet‐Rescan et al. 2021; King et al. 2020; Meza‐Joya et al. 2023; Todisco et al. 2012); experimental measurement of heat tolerance to accommodate physiological responses, and laboratory selection experiments for heat tolerance to incorporate adaptative potential (Kinzner et al. 2019); GIS tools to generate host‐plant layers to account for biotic interactions (Cerasoli et al. 2020; Parida, Hoffmann, and Hill 2015), and environmental layers to consider microhabitat features (Rödder et al. 2021); fragmentation analyses to accommodate dispersal restrictions (Koot, Morgan‐Richards, and Trewick 2022); and niche factor analyses to detect spatial patterns of climate vulnerability and exposure (Meza‐Joya et al. 2023). Reconciling inferences from distinct approaches is, however, not simple as each has its own assumptions, strengths and weaknesses, so that ambiguous or conflicting results hinder confidence (e.g., Marske, Leschen, and Buckley 2011; Meza‐Joya, Morgan‐Richards, and Trewick 2024). As rising temperatures push alpine environments uphill and novel biotic interactions become apparent, models that explicitly incorporate ecological processes might enhance the biological realism of predictions and unveil the mechanisms influencing species ranges (Briscoe et al. 2019; Urban et al. 2016).

Several approaches exist to explicitly state the processes omitted by correlative models, often called process‐based (or mechanistic) models (although this terminology differs among practitioners). By simulating the mechanisms driving range and population dynamics, such models are expected to inform more realistic projections of species' responses to climate change (Briscoe et al. 2019; Urban et al. 2016), and this is especially true for ectotherms including insects (Ponti and Sannolo 2023). While promising, process‐explicit approaches typically require extensive knowledge of the studied species to gather input data (Kearney, Shine, and Porter 2009), make their own assumptions, and their implementation often requires substantial computing power and technical expertise (Briscoe et al. 2019; Diamond 2018; Dormann et al. 2012). Hybrid models offer an exciting alternative to seize the limitations of both purely correlative and mechanistic models by allowing the use of multiple data types (Diamond 2018; Dormann et al. 2012). Range shift forecasting may be improved by simulating the processes that influence responses to climate change such as: dispersal ability and evolution of range‐limiting traits (e.g., body size) to track suitable conditions; physiological tolerances and exposure and vulnerability patterns; evolutionary potential and local adaptation to persist in place; and novel biotic interactions (Urban et al. 2016). To advance this area of modelling, classical lab and field studies (e.g., common garden and reciprocal transplant experiments) are critical for understanding key ecological, evolutionary and physiological processes (see Neupane, Larsen, and Ries 2024). Although hybrid modelling approaches may improve predictions of species responses to global warming, there is no silver bullet in ENM, and the best approach will depend on the specific questions being asked and data availability (Dormann et al. 2012; Briscoe et al. 2019).

## 6 Data Gaps, Challenges, and Opportunities

Climate change is altering species ranges in ways that go beyond current predictive ability, and we lack sufficient historical data to empirically measure range shifts in response to global heating. Ecological niche modelling offers a way to predict range shifts in the face of global warming, but requires robust data and best practices to minimise uncertainty. The harsh alpine zone poses logistical challenges for sampling (Shah et al. 2020) as most mountains are difficult to access, and the growing season is limited (especially for insects), causing data to be spatially and temporally biased. Besides, climate layers used for ENM usually have coarse spatial resolution and ignore local topoclimatic factors that create fine‐scale environmental heterogeneity (Zellweger et al. 2019), and this is relevant for alpine systems where local conditions vary extensively over short distances (De Frenne et al. 2013; Hodkinson 2005; Jump, Mátyás, and Peñuelas 2009). Accounting for this has improved microrefugia detection for alpine plants (Meineri and Hylander 2017), and this might be relevant for alpine insect populations. For instance, microclimate preferences of orthopteran species along elevational gradients (from submontane to alpine) in the Bavarian Alps (Germany) changed with elevation in response to both macro‐ and microclimatic conditions (König et al. 2024). Moreover, ENM usually neglect future land‐use changes, which might lead to biased projections and estimation of actual risks (Titeux et al. 2016). For instance, some New Zealand alpine grasshoppers are thought to have expanded their ranges downward after human removal of natural forest cover created novel open habitat (Halloy and Mark 2003), but we lack data at relevant spatial scales to account for land‐use changes when projecting range responses. Filling these major data gaps is an urgent need to better inform range forecasts.

Correlative ENM approaches can accommodate distinct sources of presence‐only data to predict range shifts. Records from museums (Neupane, Larsen, and Ries 2024) and citizen science projects (e.g., iNaturalist) provide opportunities to access locality data for a number of species, where concerns about misidentifications are addressed (Feldman et al. 2021). While these data usually come from opportunistic surveys and so have geographic biases and errors that can mislead predictions, a variety of methods can ease such limitations (Feldman et al. 2021; Neupane, Larsen, and Ries 2024). For instance, by generating target‐group absences or pseudo‐absences (Mateo et al. 2010) and using geolocation‐correction methods (Smith et al. 2023) in a way that best informs range forecasts for alpine insect species (e.g., Biella et al. 2017; Baroni and Masoero 2018; Meza‐Joya et al. 2023). Statistical approaches that accommodate sampling biases and detection errors in museum and citizen science data have mostly been developed for occupancy models (McCain and Garfinkel 2021). The resurvey of historical alpine gradients does provide attractive opportunities to empirically inform insect range shifts at fine spatial scales, and in some exceptional cases may also inform changes in the distribution of the plant species they interact with (see Kerner et al. 2023). As the robustness of such approaches depends on the equivalence between historical and modern surveys, distinct source of error due to biases in the taxonomic, spatial and temporal coverage of historical data must be considered to boost confidence (reviewed in Palmer and Hill 2017). While the generalised lack of historical datasets greatly limits the empirical study of range shifts, entomological collections represent an untapped source of data to examine alpine insect responses to climate change (see McCain and Garfinkel 2021; Meza‐Joya, Morgan‐Richards, and Trewick 2022).

Some emerging technologies have potential to overcome some of the current data gaps and strengthen ecological forecasts. Remote sensing (e.g., laser scanning, hyperspectral and thermal imaging) is advancing microclimate modelling and can help to overcome scale‐size issues for modelling range shifts (Zellweger et al. 2019). Likewise, novel free and open‐source software packages such as chelsa‐cmip6 (Karger, Chauvier, and Zimmermann 2023) allow downscaling of climate data to fine resolution that can improve detection of microrefugia under global warming (e.g., Meineri and Hylander 2017). State‐of‐the‐art algorithms may also alleviate data scarcity challenges. For instance, deep neural models for the recognition of taxonomic entities systems such as TaxoNERD (Le Guillarme and Thuiller 2022) assist the extraction of multi‐taxa information (e.g., distribution and traits) from literature, an often onerous task. Tools such as environmental DNA (eDNA) metabarcoding might increase survey capacity in terrestrial alpine environments. Unlike traditional biodiversity surveys, eDNA offers a rapid and (potentially) cost‐efficient approach for assessing biodiversity (Hassan et al. 2022; Pascher, Švara, and Jungmeier 2022). While eDNA metabarcoding has rapidly gained impetus in freshwater alpine monitoring (e.g., Mächler et al. 2021; Lim et al. 2022; Chacko et al. 2023), technical issues related to quantifying biases associated with DNA spread, limited reference databases, and capture and degradation of DNA in terrestrial systems must be considered (Hassan et al. 2022; Pascher, Švara, and Jungmeier 2022). Boosting scientific capability to envisage the future of alpine diversity requires taking full advantage of all data and tools we have at hand.

## Author Contributions

**Fabio Leonardo Meza‐Joya:** conceptualization (equal), data curation (lead), formal analysis (lead), funding acquisition (equal), investigation (lead), methodology (lead), validation (lead), visualization (lead), writing – original draft (lead). **Mary Morgan‐Richards:** conceptualization (equal), data curation (supporting), formal analysis (supporting), funding acquisition (equal), investigation (supporting), methodology (supporting), supervision (supporting), validation (supporting), writing – review and editing (supporting). **Steven A. Trewick:** conceptualization (equal), data curation (supporting), formal analysis (supporting), funding acquisition (equal), investigation (supporting), methodology (supporting), supervision (lead), validation (supporting), visualization (supporting), writing – review and editing (supporting).

## Conflicts of Interest

The authors declare no conflicts of interest.

## Supporting information


**Table S1.** List of studies examining species‐level elevational shifts for terrestrial alpine insects from online searches (Google Scholar, Web of Science).
**Table S2.** Elevational range shift estimates from studies reporting comprehensive empirical data for alpine insect species.

## Data Availability

Data available in the article's Supporting Information.
